# Bronchoalveolar lavage fluid dilution in ICU patients: what we should know and what we should do

**DOI:** 10.1186/s13054-018-2300-x

**Published:** 2019-01-24

**Authors:** Yuetian Yu, Chunyan Liu, Zhongheng Zhang, Hui Shen, Yujie Li, Liangjing Lu, Yuan Gao

**Affiliations:** 1 Department of Critical Care Medicine, Ren Ji Hospital, School of Medicine, Shanghai Jiao Tong University, 145 Middle Shangdong Road, Shanghai, 200001 China; 2 Department of Emergency, Minhang District Central Hospital, 170, Xinsong Road, Shanghai, 201100 China; 3 Department of Emergency Medicine, Sir Run Run Shaw Hospital, Zhejiang University School of Medicine, 3, East Qingchun Road, Hangzhou, 310020 China; 4 Department of Laboratory Medicine, Shanghai East Hospital, Tongji University School of Medicine, 1800, Yuntai Road, Shanghai, 200123 China; 5 Department of Rheumatology, Ren Ji Hospital, School of Medicine, Shanghai Jiao Tong University, 145, Middle Shangdong Road, Shanghai, 200001 China

The development of bronchoscopy and bronchoalveolar lavage (BAL) has led to an increase in their use in intensive care units (ICUs), where their applications for differential diagnosis of pulmonary diseases make them indispensable instruments for intensivists [1]. Despite their common use, a few studies have raised concerns about potential impacts on bronchoalveolar lavage fluid (BALF) dilution, which affects mainly the quantitative detection of soluble substances. Urea is a diffusible substance that can easily be detected in capillaries and alveolar spaces. The urea concentration in plasma and that in BALF are approximately equal and their ratio (urea plasma/urea BALF) has previously been applied as an index of BALF dilution. Furthermore, it has been shown that the ratio of high-quality lavage is low in clinical settings [2, 3].

We reviewed all ICU-admitted patients who received BAL from January 2016 to September 2018 in Ren Ji Hospital and analyzed their urea plasma/urea BALF values. Guidelines of the American Thoracic Society were followed during the BAL procedure [3]. (The procedure is described in Additional file 1.) Among 223 patients included, the median level of urea plasma/urea BALF was 4.2 (interquartile range of 3.2–8.6). The patients were categorized into groups A (urea plasma/urea BALF <4.2) and B (urea plasma/urea BALF ≥4.2). The patients in group A were more likely to receive bronchodilators (35.6% versus 15.9%, *P* <0.001) and a recruitment maneuver (15.5% versus 5.3%, *P* = 0.013) than those in group B. More invasive pulmonary aspergillosis (IPA) patients with BALF galactomannan of more than 0.5 could be detected in group A than in group B (84.6% versus 33.3%, respectively; *P* = 0.019) as well as more bacterial pneumonia patients with the quantitative cultures of BALF of more than 10^4^ CFU/mL (90.6% versus 52.7%, respectively; *P* <0.001). Primary care physicians performed more BAL than residents did (58.3% versus 31.8%, respectively), especially in group A (Table 1).

**Table 1:** Demographics and clinical characteristics of the patients

Characteristics	All patients	Group A (urea plasma/urea BALF <4.2)	Group B (urea plasma/urea BALF ≥4.2)	*P* value
	*n* = 223	*n* = 110	*n* = 113	
Age, years	54 (43–67)	51 (43–66)	56 (43–67)	0.945
Gender, male	103 (46.2)	53 (48.2)	50 (44.2)	0.556
BMI, kg/m^2^	21.9 (18.5–23.4)	22.1 (18.4–23.4)	21.8 (18.5–23.4)	0.515
PaO_2_/FiO_2_	210.4 (120.4–271.5)	250.9 (206.7–320.5)	137.4 (88.6–210.4)	<0.001
Pulmonary disease				
AECOPD	68 (30.5)	33 (30.0)	35 (30.9)	0.875
CAP	61 (27.4)	28 (25.5)	35 (30.9)	0.36
HAP	33 (14.8)	17 (15.5)	14 (12.4)	0.508
VAP	16 (7.2)	7 (6.4)	9 (7.9)	0.643
IPA	28 (12.6)	13 (11.8)	15 (13.3)	0.743
Others	17 (7.5)	12 (9.0)	5 (4.6)	0.068
APACHE II score	17 (13–23)	16 (14–22)	17 (13–23)	0.799
Intubation and mechanical ventilation	47 (21.1)	21 (19.1)	26 (23.0)	0.473
Lesion location				
Upper lobe	56 (25.1)	26 (23.6)	30 (26.5)	0.616
Middle and lower lobe	93 (41.7)	51 (46.4)	42 (37.2)	0.164
Diffusive lesions	74 (33.2)	33 (30.3)	41 (36.3)	0.319
Sedative and narcotic drugs				
Midazolam and fentanyl	96 (43.0)	51 (46.4)	45 (39.8)	0.324
Propofol and fentanyl	89 (39.9)	42 (38.2)	47 (41.6)	0.603
Dexmedetomidine	38 (17.1)	17 (15.4)	21 (18.6)	0.534
Bronchodilators was given before BAL	57 (25.6)	39 (35.6)	18 (15.9)	<0.001
RM before BAL	23 (10.3)	17 (15.5)	6 (5.3)	0.013
Operator				
Resident	71 (31.8)	5 (4.5)	66 (58.4)	<0.001
Primary care physician	130 (58.3)	93 (84.5)	37 (32.7)	<0.001
Others	22 (9.9)	12 (11.0)	10 (8.9)	0.616
Diagnosed with bacterial pneumonia	178 (79.8)	85 (77.3)	93 (82.3)	0.348
BALF GM >0.5 in IPA patients	16 (57.1)	11 (84.6)	5 (33.3)	0.019
Quantitative cultures of BALF >10^4^ CFU/mL in bacterial pneumonia patients	126 (70.8)	77 (90.6)	49 (52.7)	<0.001

Data are expressed as median (Q1–Q3) or number (percentage). *P* values for comparison between urea plasma/urea BALF ≥4.2 and <4.2 groups.

Abbreviations: *AECOPD* acute exacerbation of chronic obstructive pulmonary disease, *APACHE II* Acute Physiology and Chronic Health Evaluation II, *BAL* bronchoalveolar lavage, *BALF* bronchoalveolar lavage fluid, *BMI* body mass index, *CAP* community-acquired pneumonia, *CFU* colony-forming units, *FiO*_*2*_ fractional concentration of inspired oxygen, *GM* galactomannan, *HAP* hospital acquired pneumonia, *IPA* invasive pulmonary aspergillosis, *PaO*_*2*_ partial pressure of arterial oxygen, *RM* recruitment maneuver, *VAP* ventilator-associated pneumonia.

Pulmonary function was associated with the urea plasma/urea BALF ratio. It was found that there was a correlation between urea plasma/urea BALF and partial pressure of arterial oxygen/fractional concentration of inspired oxygen (PaO_2_/FiO_2_) (R^2^ = 0.196, *P* <0.001). The less oxygen-deficient the patient was, the lower the urea plasma/urea BALF level was (Fig. 1a,b). Sixty-eight patients with chronic obstructive pulmonary disease (COPD) were enrolled in our study. The forced expiratory volume in the first second (FEV_1_) was suggested as a measure of bronchial obstruction. FEV_1_ of less than 50% of the predicted normal value indicated the presence of severe ventilatory impairment, which led to a lower volume of instilled saline flow into the alveoli. In our study, a correlation was also found between FEV_1_ and urea plasma/urea BALF (R^2^ = 0.299, *P* <0.001). A lower value of urea plasma/urea BALF was obtained in a group with FEV_1_ of at least 50% of the predicted value than in that with FEV_1_ of less than 50% of the predicted value (*P* <0.05, Fig. 1c, d).

**Fig. 1:**
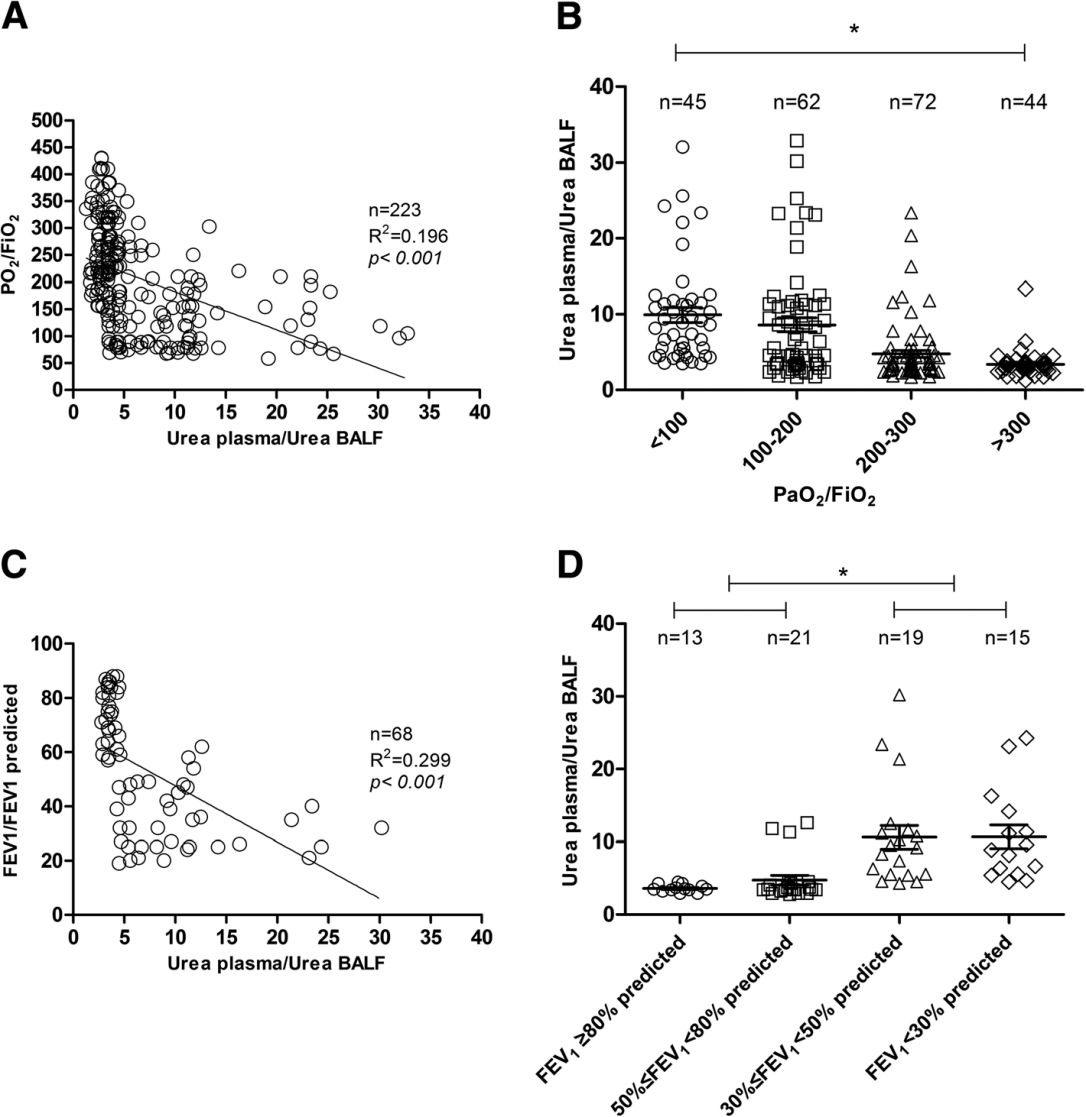
**a** Correlation between PaO_2_/FiO_2_ and urea plasma/urea BALF. **b** Comparison of urea plasma/urea BALF in different PaO_2_/FiO_2_ groups. **c** Correlation between FEV_1_/FEV_1_ predicted and urea plasma/urea BALF in patients with COPD. **d** Comparison of urea plasma/urea BALF in different FEV_1_/FEV_1_ predicted groups in patients with COPD. *P <0.05 in each group. Abbreviations: *BALF* bronchoalveolar lavage fluid, *COPD* chronic obstructive pulmonary disease, *FEV*_*1*_ forced expiratory volume in the first second, *FiO*_*2*_ fractional concentration of inspired oxygen, *PaO*_*2*_ partial pressure of arterial oxygen.

Providing appropriate training in BAL skills to intensivists while ensuring patient safety is challenging [4]. Inter-operator variability in the recovery of lavage fluid during a BAL procedure may affect the concentration of soluble substances such as galactomannan and the results of quantitative cultures [5]. More attention should be paid to patients with hypoxia and impaired pulmonary function. Bronchodilators and a recruitment maneuver may improve BALF dilution during the procedure, and residents in ICUs need more practice.

## Additional file

Additional file 1: Guidelines of the American Thoracic Society were followed during the bronchoalveolar lavage (BAL) procedure. Selection of the segment for BAL was guided by chest x-ray changes. The right middle lobe or lingual lobe was selected when diffuse infiltrates were present. Five 20-mL aliquots of sterile saline were instilled and aspirated gently in each patient. The total volume of the retrieved liquid should be greater than or equal to 30% of the total volume of the instilled saline. (ZIP 492 kb)

## Abbreviations

BAL: Bronchoalveolar lavage
BALF: Bronchoalveolar lavage fluid
FEV_1_: Forced expiratory volume in the first second
ICU: Intensive care unit
