# Supplementary material for: Bronchoalveolar lavage fluid dilution in ICU patients: what we should know and what we should do
Source: Crit Care. 2019 Jan 24;23:23. doi: 10.1186/s13054-018-2300-x (PMC6344997; doi:10.1186/s13054-018-2300-x)
Supplement: Supplementary file 1 — Guidelines of the American Thoracic Society were followed during the bronchoalveolar lavage (BAL) procedure. Selection of the segment for BAL was guided by chest x-ray changes. The right middle lobe or lingual lobe was selected when diffuse infiltrates were present. Five 20-mL aliquots of sterile saline were instilled and aspirated gently in each patient. The total volume of the retrieved liquid should be greater than or equal to 30% of the total volume of the instilled saline. (ZIP 492 kb) [file 13054_2018_2300_MOESM1_ESM.zip › supplementary material.docx]

**Supplementary materials**

Guidelines of the American Thoracic Society were followed during the bronchoalveolar lavage (BAL) procedure. Selection of the segment for BAL was guided by chest X-ray changes. The right middle lobe or lingual lobe was selected when diffuse infiltrates were present. Five 20-mL aliquots of sterile saline were instilled and aspirated gently in each patient. The total volume of the retrieved liquid should be greater than or equal to 30% of the total volume of the instilled saline.
